# Medicinal Potential of *Broussonetia papyrifera*: Chemical Composition and Biological Activity Analysis

**DOI:** 10.3390/plants14040523

**Published:** 2025-02-08

**Authors:** Ying Li, Renhua Huang, Weiwei Zhang, Qiangwen Chen, Qijian Wang, Jiabao Ye, Feng Xu

**Affiliations:** 1 College of Horticulture and Gardening, Yangtze University, Jingzhou 434025, China; liyly1219@163.com (Y.L.); wwzhangchn@163.com (W.Z.); chenqwx@163.com (Q.C.); wqjjeans@163.com (Q.W.); 2 Hubei key Laboratory of Spices & Horticultural Plant Germplasm Innovation & Utilization, Yangtze University, Jingzhou 434025, China; 3 Hubei Engineering Research Center for Specialty Flowers Biological Breeding, Jingchu University of Technology, Jingmen 448000, China; hrh510@21cn.com

**Keywords:** *Broussonetia papyrifera*, medicinal plants, flavonoids, biological activity, pharmacological effects

## Abstract

*Broussonetia papyrifera* (L.) L’Hér. ex Vent., a dioecious tree species that belongs to the Moraceae family, is a perennial plant found extensively throughout China. Its leaves are rich in essential trace elements such as copper, molybdenum, manganese, and iron, as well as various biologically active compounds, including amino acids, polysaccharides, proteins, as well as flavonoids, phenylpropanoids, and other polyphenolic compounds. These compounds exhibit significant pharmacological effects, such as antioxidant, lipid-lowering, heat-clearing, detoxifying, blood-cooling, diuretic, and immunomodulatory activities. In recent years, *B. papyrifera* has gained attention for its medicinal potential; however, breeding efforts, especially those aimed at increasing the flavonoid content, have lagged. This study reviews the progress in research on the active medicinal ingredients of *B. papyrifera*, with a focus on identification methods, classification criteria, and enrichment technologies for its medicinal components. The biosynthesis of structural genes and transcription factors in flavonoids has been investigated in *B. papyrifera*. The pharmacological effects of the secondary metabolites of *B. papyrifera* were systematically examined, aiming to offer strategies for enhancing the flavonoid content and promoting its medicinal value.

## 1. Introduction

*Broussonetia papyrifera* (L.) L’Hér. ex Vent. research indicates that *B. papyrifera* is native to China as well as subtropical regions of Southeast and East Asia. Prehistoric voyagers introduced approximately 70 species of plants to Polynesia. However, only a limited number of these plants made it to the islands of Easter Island, Hawaii, or New Zealand, with *B. papyrifera* being one of them [1]. *B. papyrifera* is a versatile tree with medicinal, edible, and feeding applications. Owing to the abundance of nutrients and active ingredients found in *B. papyrifera*, it is highly desirable for use in food and medicinal purposes. Specifically, the leaves of *B. papyrifera* are rich in various amino acids, making them an unconventional but valuable feed source for animals [2]. The cellulose-rich composition of the endodermis of the tree’s bark also makes it a premium raw material for paper production. Furthermore, *B. papyrifera* contains various bioactive compounds, including amino acids, polysaccharides, and proteins, as well as flavonoids, phenylpropanoids, and other polyphenolic compounds. Extensive pharmacological studies have demonstrated that the flavone glycosides and total flavonoids found in *B. papyrifera* can inhibit atrial contraction and protect human epidermal cells from oxidative damage caused by exposure to lead and arsenic [3]. Additionally, extracts from *B. papyrifera* possess properties such as heat clearance, detoxification [4], blood cooling, diuresis, liver cleansing, and vision improvement [5]. The leaves, in particular, contain high concentrations of glycosides, diterpenes, flavonoids, and lactones, all of which exhibit potent bioactivity [6]. The bark, leaves, and roots of *B. papyrifera* can be used to treat arthritis [7], promote wound healing and reduce skin infections, treat diarrhea [8], protect the liver [9], regulate blood sugar [10], respiratory tract infections and whiten skin [11]. Moreover, various secondary substances are present in fruits, roots, bark, and other parts of the *B. papyrifera*, making it a highly valuable resource. Owing to its rich nutritional and medicinal components, *B. papyrifera* has attracted increasing attention in recent years, leading to significant advancements in research in this field.

*B. papyrifera* contains more than 100 flavonoids, including a diverse range of compounds such as quercetin, luteolin, dihydroflavones, and liquiritigenin [12]. Some of these flavonoids have proven to be excellent inhibitors of tyrosinase [13]. Beyond their function in managing plant growth and development, flavonoids are also essential in responding to abiotic stress. Research has shown that plants produce relatively high levels of flavonoids under conditions such as excessive light, extreme temperatures, drought stress, and pathogen attack [14]. Furthermore, differences in the accumulation of flavonoids in male and female plants have been reported. Compared with male leaves, female leaves, for example, tend to have greater total flavonoid contents during the flowering stage. Under stress conditions, compared with nonstressed environments, both male and female leaves presented significantly greater total flavonoid contents during the deciduous stage [15]. These effects of *B. papyrifera* flavonoids have led to extensive research both domestically and internationally, with applications found in the fields of medicine, cosmetics, biological feed, and the chemical industry, among others.

## 2. Medicinal Active Ingredient

Numerous isolated compounds from *B. papyrifera* have been scientifically proven to possess diverse pharmacological activities, such as antibacterial, antiviral, anti-inflammatory [16], and antitumor properties [17]. In particular, flavonoid compounds derived from the root bark of *B. papyrifera* have been shown to significantly inhibit proinflammatory mediators [18]. Research has additionally demonstrated that polyphenolic substances present in *B. papyrifera* significantly inhibit the catalytic function of SARS-CoV-1 and MERS Mpro, resulting in certain antiviral effects [19]. It can improve the growth of plants under heavy metal stress by binding organic acids, carbohydrates, and proteins [20]. It is anticipated that with further advancements in the extraction process, more active medicinal ingredients can be isolated and identified from *B. papyrifera* [21].

**Figure 1:**
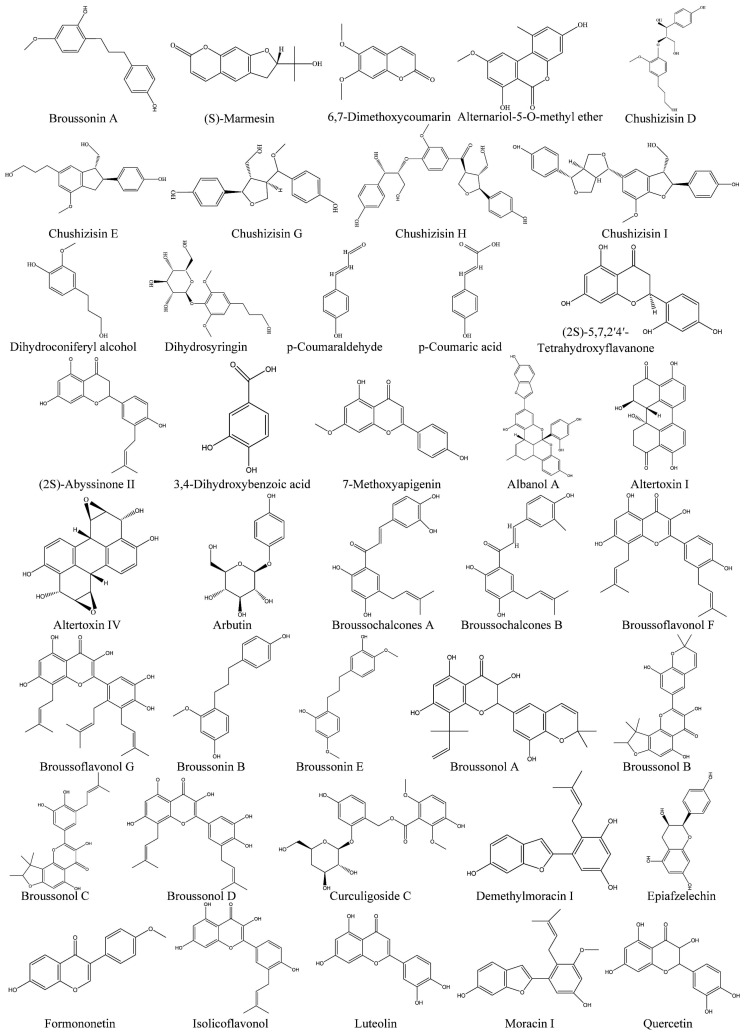
Phytochemical components identified in *B. papyrifera*.

Based on the classification of active medicinal ingredients, *B. papyrifera* can be categorized into amino acids, polysaccharides, and proteins, as well as flavonoids, phenylpropanoids, and other polyphenolic compounds (Figure 1). Through repeated column chromatography on silica gel and Sephadex LH-20, five compounds, namely, daucosterol, butei-4-methyl ester, liquiritigenin, quercetin, and dihydroquercetin, were successfully isolated from *B. papyrifera* for the first time. Spectroscopic methods were utilized to confirm their identities. Additionally, five compounds, namely, apigenin, cosmosiin, luteolin, vitexin, and scopoletin, were separated from the leaves of *B. papyrifera* via chromatography and recrystallization techniques. Furthermore, phenylpropanoids, terpenes, and flavonoids, specifically D-galactitol, ergosterol peroxide, and black fat, as well as six flavonoids and two triterpenes, were discovered in the bark of *B. papyrifera*. Compounds such as ethyl palmitate and linoleic acid, as well as palmitic acid and phytoalcohol, are isolated from *B. papyrifera*. Ethyl palmitate and linoleic acid were isolated from the fruit of *B. papyrifera*, along with compounds such as palmitic acid and phytol [22].

### 2.1. Polyphenolic Compounds

Polyphenolic compounds, such as phenolic acids and anthocyanins, are characterized by multiple phenolic hydroxyl groups attached to aromatic rings. Recent research has revealed the various pharmacological properties of polyphenolic compounds, including antioxidant, blood glucose-lowering, antitumor, anti-inflammatory, and antibacterial effects. *B. papyrifera* contains approximately 25 types of polyphenolic compounds [23] (Table 1). Zhuang et al. identified several active compounds from *B. papyrifera* and, through an in vitro aromatase inhibition assay, confirmed that five of these compounds are polyphenolic [24]. Isolation of tyrosinase from *B. papyrifera* leaves with inhibitory effects on both monophenolases and diphenolases [25]. Zhou et al. isolated thirteen known phenolic compounds and two new compounds from *B. papyrifera* fruits via ethanol extraction. The neuroprotective effects of these compounds were demonstrated through a DPPH free radical scavenging assay and H_2_O_2_-induced damage in SY5Y cells, especially at a concentration of 20 mM [22]. Polyphenolic compounds from *B. papyrifera* act as inhibitors of elevated nitric oxide synthesis. These findings suggest that polyphenolic compounds in *B. papyrifera* may be beneficial for treating inflammation [26]. Additionally, Li et al. identified two polyphenolic compounds from *B. papyrifera*, and subsequent studies have reported a variety of such compounds [27]. Moreover, numerous phenylpropanoids have also been identified in *B. papyrifera*.

Phenylpropanoids, which are widely present in the plant kingdom, play significant roles as essential components of various structural polymers. They provide protection against ultraviolet (UV) light, herbivores, and pathogens while also participating in plant–pollinator interactions as anthocyanins and odor compounds. According to statistics, approximately 30 types of phenylpropanoids [28], including 12 lignan compounds from *B. papyrifera* fruits, have been identified in recent years. Some of these compounds have shown antioxidant activity against H_2_O_2_-induced damage in PC12 cells with high differentiation, whereas others have demonstrated DPPH radical scavenging activity [29]. First isolated six compounds, such as dihydroconiferyl alcohol, erythro-1-(4-hydroxyphenyl)glycerol, and threo-1-(4-hydroxyphenyl)glycerol, from the ethanol extract of *B. papyrifera* fruit [22]. Yang et al. discovered four compounds with strong antioxidant activity from the *n*-butanol extract of *B. papyrifera*, suggesting the potential of these compounds as aromatase modulators from natural sources [30]. Malanik et al. isolated 14 compounds in young branches of *B. papyrifera* with ethanol extract, including two previously unknown phenylpropanoids, which exhibited anti-inflammatory and antioxidant activities in cells [31]. Li et al. isolated four types of lignans and eight types of coumarins from bark extracts, along with two new compounds from leaves [27] (Table 2).

### 2.2. Terpenes and Alkaloids

Terpenoids are the largest and most structurally diverse class of naturally occurring compounds. Terpenoids are crucial for plant growth, development, defense systems, and interspecific competition [32]. They are widely used as fragrances, condiments, and cosmetics, with notable examples being menthol and perillyl alcohol.

Terpenoid biosynthesis involves two main synthetic pathways: the mevalonate (MVA) pathway and the 1-deoxy-D-xylulose-5-phosphate (DXP) pathway. Prenyl diphosphate serves as a key intermediate in both [33]. The MVA pathway primarily occurs in the cytoplasm and begins with acetyl-CoA. Through a series of enzymatic reactions, this pathway produces mevalonate, which is then phosphorylated and decarboxylated to generate isopentenyl diphosphate (IPP) and dimethylallyl diphosphate (DMAPP). It is primarily responsible for the synthesis of sesquiterpenes, triterpenes, and sterols [34]. The DXP pathway primarily occurs in the chloroplast, commencing with pyruvate and glyceraldehyde-3-phosphate. Through a series of enzymatic reactions, it produces isopentenyl diphosphate (IPP) and dimethylallyl diphosphate (DMAPP). This pathway is chiefly responsible for the synthesis of monoterpenes, diterpenes, and tetraterpenes [35]. Previous studies have identified seventeen terpenoids from *B. papyrifera* [36] (Table 3), including three compounds with inhibitory effects on tyrosinase and xanthine oxidase, making them potential ingredients for skin care products [37]. Song et al. identified two known terpenoids squalene and butyrospermol acetate, in *B. papyrifera* [38], Additionally, four new triterpenoid compounds were discovered in the ethanol extract of *B. papyrifera* [36].

Alkaloids are natural compounds that produced by plants, bacteria, fungi, and other organisms. These compounds are used to treat antimicrobial, antimalarial, anticancer, arrhythmia, and antiasthmatic. Wang et al. reported that alkaloids from *Fritillaria* species exhibit strong antitumor activity [39]. *Zanthoxylum* L. alkaloids have antibacterial properties and can cure hepatitis B virus [40], whereas that *Dendrobium* species exhibit neuroprotective, anti-inflammatory, and antitumor activities [41]. Alkaloids from *Gelsemium elegans* show antifungal activity [42], highlighting the significant research potential of plant-derived alkaloids. However, research on alkaloids from *B. papyrifera* is limited, with 11 alkaloids isolated from this plant to date [43] (Table 4).

### 2.3. Other Compounds

In addition to the active ingredients mentioned above, *B. papyrifera* contains approximately 45 other compounds, including steroids, sterols, arylpropynes, and lactones (Table 5). Yadav reported 8 compounds [43], whereas Qureshi et al. identified 4 compounds [28], and isolated 15 compounds in 2019 [27], and reported four compounds in the *n*-butanol extract that effectively inhibited estrogen biosynthesis in human ovarian granulosa cells [30].

## 3. Metabolism of Flavonoids in *B. papyrifera*

The medicinal properties of *B. papyrifera* are attributed primarily to its bioactive constituents, including flavonoids, terpenes, and polyphenols. Among these, flavonoids have received the most extensive research attention. Flavonoids are a class of natural compounds distinguished by their C6-C3-C6 skeleton or phenylpropylpyran structure. It is widespread in different plant tissues, such as roots, stems, leaves, flowers, and fruits [44]. Flavonoids can be classified into different subgroups according to the degree of oxidation of the central heterocycle, including flavones, flavonols, flavanols, isoflavones, flavanones, chalcones, and anthocyanins [45]. These compounds often occur in modified forms in plants, such as through hydroxylation, methylation, acylation, and glycosylation. Glycosylated flavonoids are the most prevalent derivatives found in nature [46]. There are over 9000 different flavonoids in plants [47], and *B. papyrifera* alone contains 192 flavonoids, with a high concentration in its leaves. Flavonoids play crucial roles in plant growth, development, and response to various stressors, both biotic and abiotic [48]. They offer protection against ultraviolet radiation [49], increase salt-alkali tolerance, promote drought resistance, and improve adaptation to low temperatures [50]. In addition to their importance in plants, flavonoid extracts have multiple medicinal activities, such as protecting against oxidative cell damage, inhibiting platelet aggregation, scavenging free radicals, and treating cardiovascular and cerebrovascular diseases, Alzheimer’s disease, and cancer [51]. *B. papyrifera* exhibits significant amplification of four key enzyme families involved in flavonoid synthesis, exceeding those of other plants, such as *B. papyrifera*, poplar, *Arabidopsis*, and peach. This likely contributes to its flavonoid ccumulation and superior disease immunity [52]. Jiao et al. identified 192 flavonoids from *B. papyrifera*, including 46 flavonols, 23 flavonoid carbon glycosides, 10 flavan-3-ols, 9 anthocyanins, and dihydroflavones. There were six species of dihydroflavones, seven species of dihydroflavonols, four species of isoflavones, and two species of chalcones. The flavonoid content of *B. papyrifera* is influenced by sex and developmental stage [15]. This section reviews the research on the biosynthesis and transcriptional regulatory mechanism of flavonoids in *B. papyrifera*, with a focus on flavonoid biosynthetic pathways, related enzyme-encoding genes, and flavonoid synthesis-related transcription factors.

### 3.1. Flavonoid Biosynthetic Metabolic Pathways and Related Enzyme-Encoding Genes

**Figure 2:**
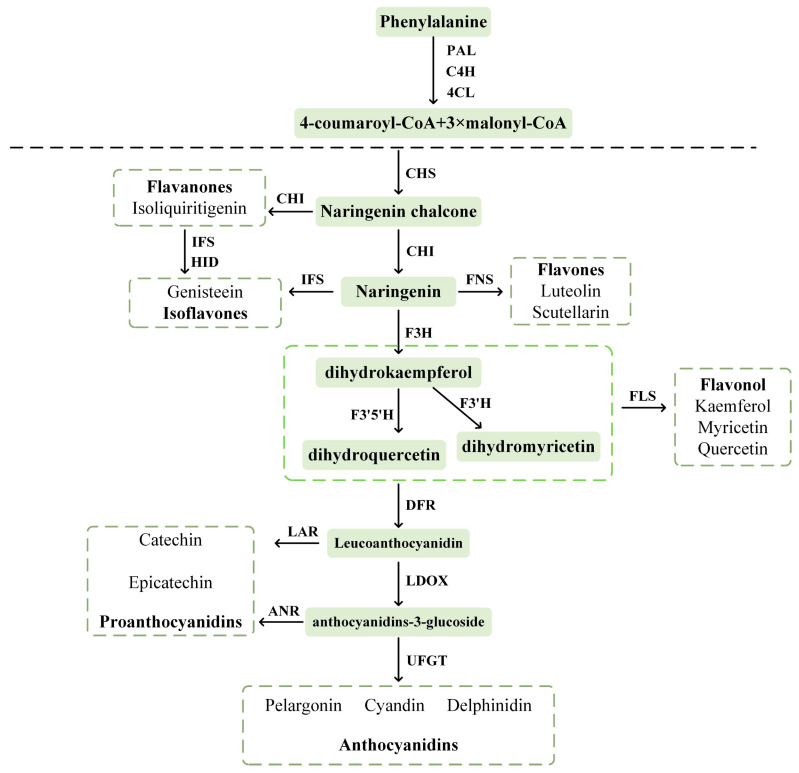
Schematic diagram of flavonoid metabolic pathway. Abbreviations: PAL, phenylalanine ammonia-lyase; C4H, cinnamic acid 4-hydroxylase; 4CL, 4-coumarate CoA ligase; CHS, chalcone synthase; CHI, chalcone isomerase; IFS, isoflavone synthase; FNS, flavone synthase; F3H, flavanone 3-hydroxylase; F3′H, flavonoid 3′-hydroxylase; F3′5′H, flavanone 3′,5′-hydroxylase; FLS, flavonol synthase; DFR, dihydroflavonol-4-reductase; LAR, leucoanthocyanidin reductase; LDOX, leucoanthocyanidin dioxygenase; ANR, anthocyanidin reductase; UFGT, uridine diphosphate-glucose: flavonoid 3-O-glucosyltransferase.

Flavonoids are present in all parts of *B. papyrifera* [53]. The synthesis of flavonoids in *B. papyrifera* has been extensively studied. The upstream pathway of flavonoid biosynthesis begins with phenylalanine ammonia-lyase [54] (Figure 2).

Coumaroyl-CoA serves as the precursor substance for the metabolic pathway of flavonoid biosynthesis. Phenylalanine is initially produced through the shikimate metabolic pathway and then undergoes a three-step enzymatic reaction in the phenylpropanoid pathway to produce coumaric acid. Coumaric acid is then converted into coumaroyl-CoA, which is the precursor of the flavonoid biosynthetic pathway, through the action of 4-coumarate:CoA ligase [55]. The conversion of coumaroyl-CoA and malonyl-CoA results in the synthesis of chalcone-by-chalcone synthase. Chalcone isomerizes further to convert chalcone into naringenin. Various types of flavonoids are produced through the action of different enzymes, such as flavone synthase for flavone synthesis, isoflavone synthase for isoflavone synthesis, and flavanone-3-hydroxylase for the synthesis of isoflavones [56]. Dihydroflavonols are synthesized under the action of flavonol synthase, which further produces flavonols. Alternatively, dihydroflavonol-4-reductase can be involved in dihydroflavonols to colorless anthocyanins. These colorless anthocyanins can be converted into a variety of anthocyanins through anthocyanidin synthase or into flavanols through the action of leucoanthocyanidin reductase [57]. The genes *FLS* and *CHS*, which are associated with the anabolic pathway of flavonoids, were initially isolated from *Petroselinum hortense* [58]. These key enzyme-encoding genes have also been identified in apple, citrus [59], poplar, and *Arabidopsis* [60]. During the fruit and leaf drop stages of *B. papyrifera*, the expression patterns of *CHI* and *DFR*, as well as *CHS1* and *HCT4*, were opposite in female and male plants. During this stage, various compounds, including phlorizin, dihydrokaempferol, garbanzol, epigallocatechin, epiafzelechin, catechin, and afzelechin, accumulate [15].

Studies have indicated that the levels of quercetin and myricetin in the roots of *B. papyrifera* increase under cadmium stress, whereas the activity of enzymes involved in flavonoid synthesis decreases [61]. *B. papyrifera* thrives in cadmium (Cd)-contaminated areas, demonstrating enhanced flavonoid metabolism, inhibited lignin biosynthesis, and associations with symbiotic fungi such as *Rhizophagus irregularis* (arbuscular mycorrhizal fungi, AMF) [62]. This study examined the performance of *B. papyrifera* both with and without inoculation of *R. irregularis* under Cd stress. The results indicated that Cd stress significantly increased the levels of eriodictyol, quercetin, and myricetin, while *R. irregularis* further elevated the levels of eriodictyol and genistein but decreased rutin content. Lignin levels remained unchanged. Additionally, 26 co-regulated genes were identified, among which *BpC4H2*, *BpCHS8*, and *BpCHI5* were closely associated with eriodictyol biosynthesis, underscoring their critical role in enhancing *B. papyrifera’s* tolerance to Cd stress [63].

The transcriptome of *B. papyrifera* consists of 141 genes associated with the phenylpropanoid metabolism pathway and 41 genes related to the flavonoid biosynthesis pathway. This study revealed that fluctuations in flavonoid content are linked to the expression patterns of flavonoid-related genes. Genes involved in energy and glucose metabolism, polysaccharide metabolism, cytoskeletal metabolism, signal transduction, and protein and amino acid metabolism may influence the growth and development of *B. papyrifera*, thereby influencing its flavonoid content [14].

### 3.2. Transcription Factors Related to Flavonoid Biosynthesis in B. papyrifera

In the plant kingdom, transcription factors integrate to the promoters of flavonoid structural genes and regulate their transcription, essentially participating in flavonoid biosynthesis. Currently, studies have shown that MYB, among other transcription factors, plays an important role in the biosynthesis of flavonoids [64].

**Figure 3:**
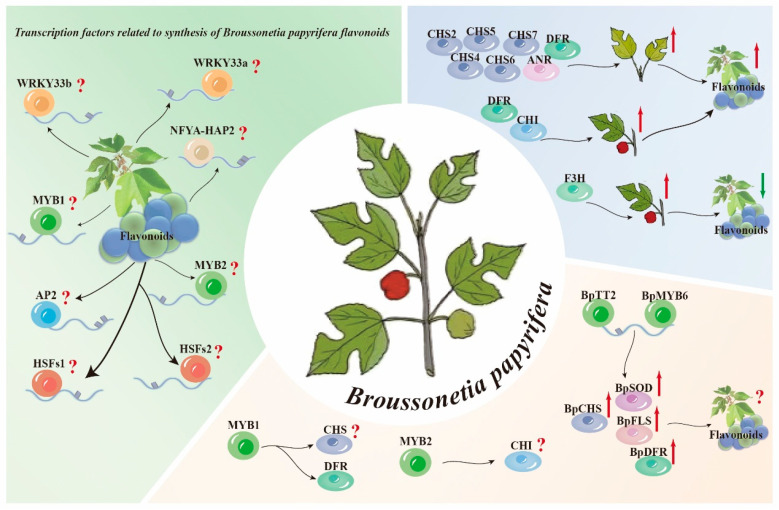
Schematic diagram of the biosynthesis pattern of flavonoids in *B. papyrifera.* (“⬆” Indicates addition or positive regulation,“⬇” Indicates reduction or negative regulation, “?” Indicates unknown).

Extensive research has been conducted on Moraceae plants to understand the regulation of flavonoid synthesis by transcription factors. It has been reported that in *Morus alba* L., MaMYB4 positively regulates *FLS* and negatively regulates *ANS* [65]. In mulberry, *MnMYBJ* and *MnMYB4* negatively regulate anthocyanin biosynthesis, whereas *MnMYB330* functions as a positive regulator. These findings highlight the multifaceted regulatory functions of MYB transcription factors in flavonoid pathways across various species. By overexpressing *BpTT2* and *BpMYB6* in *B. papyrifera*, the transcription of downstream *BpCHS1* and *BpDFR1* is effectively increased, resulting in improved antioxidant enzyme activity and increased accumulation of polyphenolic compounds in the plant [65]. Eight transcription factors, including two MYB transcription factors, *MYB1* and *MYB2,* associated with *DFR*, *CHS*, and *CHI*, respectively, were identified from two key modules closely linked to flavonoids. Furthermore, high expression of the *B. papyrifera DFR* and *CHI* genes promoted flavonoid accumulation, whereas inhibiting the expression of *F3H* genes increased flavonoid synthesis. Coexpression network analysis revealed that eight transcription factors (*HSFs1* and *AP2*) are related to *B. papyrifera* flavonoid synthesis. However, knowledge regarding flavonoid synthesis genes in *B. papyrifera* is limited, with relevant studies showing significant expansion of only four key enzyme families, including *CHS* and *DFR*, in the flavonoid synthesis pathway [15] (Figure 3).

## 4. Flavonoid Extraction Method

Various active substances are found in different parts of *B. papyrifera*, including the roots, stems, leaves, and fruits. These substances include phenolic compounds, flavonoids, lignans, terpenoids, alkaloids, and fatty oils. This difference suggests that flavonoid metabolism plays a significant role as a secondary pathway in plants. Furthermore, certain flavonoids found in *B. papyrifera*, such as broussochalcone A, papyriflavonol A, and 3′-(3-methylbut-2-enyl)-3′,4′,7-trihydroxyflavane, have shown potential as drugs against COVID-19 [19]. Current investigation on *B. papyrifera* mainly focuses on stress resistance, nutrition, and pharmacological ingredients. Studies have revealed the isolation of more than one hundred flavonoids from *B. papyrifera* [66] (Table 6), including vitexin, quercetin, apigenin, luteolin, isoliquiritigenin, and galangal.

Common methods used to extract *B. papyrifera* flavonoids include solvent extraction, microwave-assisted extraction, ultrasonic-assisted extraction, and other techniques to measure the total flavonoid content of *B. papyrifera* [67] (Table 7). Solvent extraction is a traditional method that relies on the solubility of flavonoid compounds. The choice of solvent depends on the solubility of flavonoids and the impurity level [68]. Inorganic solvents are used to dissolve flavonoids in alkaline solutions, which are subsequently acidified to precipitate the flavonoids. On the other hand, organic solvents with varying polarities are selected for the extraction of target components and removal of impurities. While solvent extraction is easy to use, it is difficult and time-consuming to recover [69]. Ultrasonic-assisted extraction is often employed in conjunction with solvent techniques to increase the solubility of flavonoid compounds and preserve their structure. This method is efficient and rapid [70].

Microwave-assisted extraction involves heating medicinal materials and solvents via a microwave reactor to extract active ingredients. This method offers advantages such as rapid heating, easy control, safety, environmental friendliness, high efficiency, and energy savings [71].

Ionic liquids, a new class of environmentally friendly solvents, have been widely utilized in various industries [72]. Recent research has shown that benzothiazole methane sulfonate, an ionic liquid, can be used as an extractant in conjunction with ultrasonic-assisted extraction to efficiently extract total flavonoids from *B. papyrifera*. Through optimization of the extraction conditions, it was found that with an ionic liquid concentration of 0.5 mol/L, 60% ethanol, a solid-liquid ratio of 1:20, and extraction at 60 °C for 20 min, the total flavonoid content in *B. papyrifera* was 0.4685 mg/g [73]. In contrast when a traditional extraction process without auxiliary conditions such as ultrasonication was used, the total flavonoid content was 37.33 mg/g when the ethanol concentration was 30%, the extraction time was 2.5 h, the temperature was 69 °C, and the solid-liquid ratio was 9:100 [14]. These results show that the most effective process for extracting and purifying flavonoids from leaves involves 50% ethanol, a solid-liquid ratio of 1:30, ultrasonic extraction at 50 °C for 40 min, filtration, and concentration, extraction with petroleum ether to remove fat-soluble impurities such as chlorophyll, and acidification of water with hydrochloric acid. The ethyl acetate was subsequently used to extract the aqueous phase; the ester layer was then separated and dried in a vacuum, resulting in a total flavonoid content of 35.36% [74].

Using response surface methodology (RSM) [70], the ultrasonic-assisted extraction of flavonoids from *B. papyrifera* leaves was optimized, resulting in total flavonoid content of 37.946 mg/g under the following conditions: 69% ethanol, 37 min of ultrasonication, and a material-to-liquid ratio of 1:52. Another experiment utilizing ethanol reflux extraction, which was also optimized via RSM, yielded 23.93 mg/g total flavonoids under the conditions of 70% ethanol, a solid-to-liquid ratio of 1:16, an extraction temperature of 75 °C, and an extraction time of 117 min [75]. By modifying the total flavonoid reflux extraction process used for *Sorbus tianschanica*, a yield of 55.14 mg/g total flavonoids from *B. papyrifera* was obtained via 90% ethanol, a solid-to-liquid ratio of 1:35, an extraction temperature of 85 °C, and an extraction time of 80 min [76].

Furthermore, microwave-assisted extraction of total flavonoids from *B. papyrifera*, optimized through single-factor and orthogonal tests, yielded a total flavonoid content of 79.63 mg/g when using 55% ethanol, a microwave duration of 15 min, a power setting of 450 W, and a solid-to-liquid ratio of 1:12 [77].

**Table 7:** Flavonoids extraction methods of *B. papyrifera*.

No.	Method	Extraction Conditions	Advantages and Disadvantages	Total Flavonoids	Reference
1	Ultrasonic-assisted ionic liquid extraction method.	Ionic liquid concentration 0.5 mol/L; ethanol concentration 60%; solid–liquid ratio 1:20; 60 °C; extraction 20 min.	Advantages: efficient extraction, short extraction time, environmentally friendly. Disadvantages: high cost, potential toxicity.	0.4685 mg/g	[73]
2	Ultrasound-assisted method.	Sample powder 10 g; cold soak in 70% ethanol; ultrasonic for 20 min; power 300 W; recovery under reduced pressure; elution with 70% ethanol.	Advantages: short extraction time, little solvent, simple operation. Disadvantages: high equipment cost, solvent selection limit.	2.18%	[74]
3	Ultrasound-assisted method.	Ethanol concentration 50%; material–liquid ratio 1:30; ultrasonic extraction at 50 °C for 40 min.		35.36%	[74]
4	Ultrasound-assisted method.	The ethanol concentration is 69%; the ultrasonic time is 37 min; the material-to-liquid ratio is 1:52.		37.946 mg/g	[78]
5	Alcohol extraction method.	Ethanol concentration 70%; material–liquid ratio 1:16; extraction temperature 75 °C; extraction time 117 min.	Advantages: low cost, wide scope of application, efficient extraction. Disadvantages: the extraction time is longer, solvent residue problem.	23.93 mg/g	[75]
6	Alcohol extraction method.	Ethanol concentration 90%; solid–liquid ratio 1:35; extraction temperature 85 °C; extraction time 80 min.		55.14 mg/g	[76]
7	Microwave-assisted extraction method.	Ethanol concentration 55%; microwave time 15 min; power 450 W; material–liquid ratio 1:12.	Advantages: efficient and fast, extract efficiency, energy-saving, and environmental protection. Disadvantages: high equipment cost, potential component degradation.	79.63 mg/g	[77]
8	Spectrophotometry.	Sample powder 2 g; petroleum ether 100 mL; degreasing with Soxhlet extractor for 6 h; Soxhlet extraction with 100 mL 60% methanol for 6 h; measurement at 505 nm.	Advantages: low cost, simple operation, high sensitivity. Disadvantages: poor selectivity, unable to provide structural information.	6.05%	[79]
9	Spectrophotometry.	5 g sample powder; extraction with 50 mL petroleum ether; 50 mL 95% ethanol; condensation reflux; measurement at 500 nm.		3.13%	[79]
10	Spectrophotometry.	Grind in 2 mL of concentrated HCl and ethanol; centrifuge at 12,000× *g* for 10 min; soak the supernatant in 80 °C water for 10 min; measure at 270, 300, and 330 nm.			[14]

In conclusion, these studies demonstrate that these techniques offer significant advantages over traditional methods. Specifically, the optimization of ethanolic extraction and microwave-assisted extraction via the Box–Behnken design in response surface methodology for flavonoids from *B. papyrifera* leaves provides simpler and more efficient processes. These techniques are easy to perform, cost-effective, provide higher yields, and have better stability. These results provide important insights into the manufacturing and utilization of flavonoids derived from the leaves of *B. papyrifera*. Moreover, combining various extraction techniques can greatly increase the extraction efficiency of flavonoid compounds from *B. papyrifera*.

## 5. Pharmacological Effects

### 5.1. Antitumor

This study revealed that active compounds from *B. papyrifera* can be utilized to treat human bladder cancer, establishing their potential medicinal use. By examining cell proliferation, apoptosis, and autophagy, the cytotoxic effects of these compounds were evaluated. These results indicate that the plant constituents have cytotoxic effects on human bladder cancer cells, including cisplatin-resistant T24R2 cells. This finding presents a potential source for developing anticancer drugs and provides new treatment options for bladder cancer patients [43]. Studies have demonstrated that extract from the bark of *B. papyrifera* can induce DNA fragmentation associated with apoptosis, increase the accumulation of sub-G1 phase cells, and inhibit the proliferation of HT-29 colon cancer cells. It can also significantly enhance the expressive capabilities p53, caspase-3, and Bax in HT-29 cells. Moreover, the partial *n*-butanol extract inhibited the production of nitric oxide by suppressing iNOS expression in macrophages [80]. Studies have shown that the ethanol extract from *B. papyrifera* disincentives the proliferation of MG63 human osteosarcoma cells and is associated with apoptosis and cell cycle arrest [81].

Broussoflavonol B, which is isolated from *B. papyrifera*, effectively inhibited the growth of estrogen receptor (ER)-negative breast cancer SK-BR-3 cells at submicromolar concentrations. Broussoflavonol B more potently inhibited growth and induced differentiation of stemlike SK-BR-3 cells compared to the anti-estrogen tamoxifen [82]. Separate multiple compounds from *B. papyrifera* bark extract showed significant antiproliferative effects on ER-positive MCF-7 cells in vitro. Broussochalcone A and broussoflavonol B effectively inhibit tumor development by reducing ERK phosphorylation. Western blotting results indicate that these compounds can substantially downregulate the secretion of estrogen receptor α. By inactivating the ERK and AKT pathways, Dou et al. demonstrated that PBPs can induce mitochondria-mediated apoptosis in HepG2 cells. This process additionally results in a heightened generation of reactive oxygen species (ROS) within the cells and a reduction in intracellular superoxide dismutase (SOD) levels [83].

The potential of kazinol Q to induce DNA cleavage in the presence of Cu. These findings indicate that the survival rate of gastric cancer SCM-1 cells is significantly reduced [84]. Broussochalcone A exhibited significant cytotoxic effects on the human liver cancer cell lines SK-Hep1 and HepG2. It regulates the cell cycle by expanding FOXO3, resulting in cell cycle arrest and the activation of proapoptotic proteins [85]. Additionally, a concentration of 20 µM broussochalcone A exhibited notable cytotoxicity against colon and liver cancer cells through the activation of β-catenin phosphorylation [86].

Meerson et al. examined the impact of flavonoids on the proliferation of mouse epidermal stem cells in vitro. Their research revealed that the suppression effect of flavonoids on cell proliferation was concentration-dependent. When the concentration of flavonoids was between 5 and 10 μg/mL, the inhibition rate of epidermal stem cells reached 50%. Throughout this timeframe, the dosage of flavonoids displayed a positive correlation with their inhibitory effect on cells [87]. In 2022, Wang et al. discovered that marmesin can induce cytotoxicity in esophageal cancer cells by restraining the PI3K/Akt pathway [88].

### 5.2. Antioxidants

Oxidative stress poses a threat to cells, DNA, and other substances, emphasizing the importance of antioxidants in protecting individuals. A variety of antioxidant activity assays have been employed to evaluate different aspects, including DPPH scavenging activity [89], and the activity of scavenging hydrogen peroxide [90], the scavenging activity for hydroxyl radicals [91], FRAP analysis [92], inhibition of lipid peroxidation, and assays for mitochondrial swelling, flavin oxidase suppressing activity, and superoxide anion radical scavenging activity [93].

The antioxidant activity of *B. papyrifera* seed oil was evaluated via the DPPH and pyrogallol autoxidation methods. The results revealed a high inhibition rate of 93.56% for hydroxyl free radicals but no significant effect on superoxide anions. Broussochalcone A, which was isolated from the bark of *B. papyrifera* by Sun et al., has been identified as a potent natural antioxidant. It effectively scavenges free radicals and inhibits the degradation of IkB in RAW 264.7 macrophages, thereby suppressing the LPS-induced expression of the iNOS protein [94]. The research investigated the antioxidant potential of crude extracts from *B. papyrifera* using both ethanol and water. The ethanol extract exhibited greater free radical scavenging activity at 5 mg/mL, the DPPH content was 62.88%, while the chelating activity of Fe^2+^ was 61.15% at 6 mg/mL [95]. The study demonstrated the significant antioxidant activity of erythro-1-(4-hydroxy-3-methoxyphenyl)-2-{4-[(E)3-hydroxy-1-propenyl]-2-methoxyphenoxy}-1,3-propanediol, which was isolated from *B. papyrifera*, through MTT and DPPH experiments [29]. Broussochalcone A and 3,4-dihydroxyisolonchocarpin, which are extracted from the roots of *B. papyrifera*, presented the highest antioxidant activity within the dose range of 0.1~1000 µM [96]. Broussoflavonol A, 5,7,3,4-tetrahydroxy-3-methoxy-8,5-diprenylflavone, and kazinol M, which were extracted from the ethanol extract of *B. papyrifera* by Malanik et al. also demonstrated good antioxidant activity [31].

### 5.3. Anti-Inflammatory Effects

Broussochalcone A from *B. papyrifera*, at a dose of 1–20 µM, has been discovered that the production of nitric oxide in macrophages activated by LPS is reduced by the suppression of IkBα phosphorylation, degradation of IkBα, and expression of iNOS [97]. Papyriflavonol A, which was isolated from *B. papyrifera* by Kwak et al. effectively inhibited human group IIA- and group V-secreted phospholipase A2, leading to a significant reduction in IgE-dependent passive skin allergic reactions in rats. These findings form the basis for developing new anti-inflammatory drugs [98]. Broussonin E has demonstrated that certain treatments can effectively manage inflammatory diseases through multiple mechanisms. One significant approach involves the inhibition of the ERK and p38MAPK pathways, both of which play crucial roles in the inflammatory response. By targeting these pathways, the treatment can diminish inflammatory signaling and contribute to reduced disease severity. Additionally, the enhancement of the JAK2-STAT3 signaling pathway further supports the body’s ability to regulate inflammation [99].

Furthermore, a range of research studies has revealed the noteworthy anti-inflammatory properties of the methanol extract derived from *B. papyrifera* when tested on RAW 264.7 cells. These studies indicate that this extract possesses a remarkable capacity to impede the synthesis of nitric oxide (NO) and the production of proinflammatory cytokines [100]. In addition, also isolated active ingredients from the ethanol extract of *B. papyrifera* [31]. Broussoflavonol H, which is isolated from *B. papyrifera*, significantly inhibits Jurkat-induced cellular IL-2 production induced by PHA and PMA [101]. Ryu et al. isolated flavanone, broussoflavanonol A, broussoflavonol B, kazinol V, kazinol W and broussochalcone C from *B. papyrifera* by 100% methanol extraction. These compounds have shown strong anti-inflammatory effects by downregulating the expression of iNOS in RAW 264.7 cells [23]. In summary, the underlying mechanism of the anti-inflammatory effects of *B. papyrifera* is primarily inhibition of NO production and iNOS expression, primarily seen in LPS or cytokine-induced macrophages, leading to increased levels of NO as a proinflammatory mediator.

### 5.4. Antibacterial and Antiviral Agents

Studies have indicated that *B. papyrifera* extract possesses antibacterial properties against *Enterococcus faecalis* [102], *Vibrio cholerae*, *Bacillus subtilis*, *Pseudomonas aeruginosa*, and other bacterial strains [103]. The hexane extract of mulberry seeds has significant inhibitory activity against *Staphylococcus aureus*, *Proteus vulgaris*, *Bacillus cereus*, *Enterobacter aerogenes*, and other bacteria. This suggests that hexane extracts have great potential as antibacterial agents against these specific pathogens. However, this study showed that this extract had no inhibitory effect on fungal strains [104]. Han et al. (2016) purified three components, BPP-1 and BPP-3, from the fruit of *B. papyrifera* and conducted in vitro antibacterial tests via the filter paper disk agar diffusion method. The findings indicated that BPP-3 demonstrates significant antibacterial properties against both Gram-positive and Gram-negative bacteria, showing diverse antibacterial effectiveness among the various constituents of BPP. Notably, BPP-3 showed marked antibacterial action against *Escherichia coli*, *Pseudomonas aeruginosa*, *Bacillus subtilis*, and *Staphylococcus aureus* [105].

Moreover, the study investigated the antibacterial activity of papyriflavonol A at various doses ranging from 1 to 1000 µM. The results demonstrated strong inhibitory effects [106], particularly against PLpro. Further research suggested that PLpro may serve as a potential anti-COVID-19 drug [19]. They extracted the flavonoids daphnegiravan F and 5,7,3′,4′-tetrahydroxy-3-methoxy-8,5′-diprenylflavone from the ethyl acetate part of *B. papyrifera*, which are related to Gram-positive and Gram-negative bacteria [16].

### 5.5. Antidiabetic

Diabetes is a long-term condition marked by either insufficient insulin production or resistance to insulin. A variety of in vitro and in vivo research studies have illuminated the potential antidiabetic effects of different extracts from *B. papyrifera*. Particularly, an investigation by Ryu et al. revealed that compounds such as broussochalcone A, papyriflavonol A, broussochalcone B, kazinol A, kazinol B, and 8-(1,1-dimethylallyl)-5-(3-methylbut-2-enyl)-3,4,5,7-tetrahydroxyflavonol show inhibitory action against α-glucosidase [107]. The study found that inhibiting the NF-kB pathway in pancreatic β-cells using kazinol U may diminish cellular damage, indicating possible therapeutic strategies to postpone the destruction of pancreatic β-cells in individuals with type 1 diabetes [108]. Lee et al. reported that *B. papyrifera* root extract improved high-fat diet-induced C57BL6 mice by activating AMPK in 3T3-L1 adipocytes. They also observed a suppression of the proinflammatory response with broussoflavonol B and kazinol J at doses of 0–100 µg/mL. These findings indicate the antidiabetic effects of the extract [10]. Protein tyrosine phosphatase 1B (PTP1B) is an important enzyme involved in the dephosphorylation process of the insulin receptor, a mechanism that contributes to insulin resistance development. Inhibitors of PTP1B have shown promise as therapeutic options for managing type 2 diabetes [109], and several compounds from *B. papyrifera*, including (1,1-dimethylallyl)-5-(3-methylbut-2-enyl)-3,4,5,7-tetrahydroxyflavonol, 3,3,4,5,7-pentahydroxyflavone, and broussochalcone A, exhibit inhibitory activity against PTP1B [109]. Research indicates the possible application of *B. papyrifera* in managing diabetes.

### 5.6. Other Pharmacological Effects

Except for pharmacological activities discussed above, the active ingredients in *B. papyrifera* also have various other effects, such as anti-gout effects, liver protection, treatment of bone diseases, anti-angiogenic activity, anti-cholinesterase effects, whitening, anti-wrinkle effects, promotion of hair growth, and immune stimulation. Broussochalcone A and 3,4-dihydroxyisoonchocarpin, both of which are found in ethanol extracts of *B. papyrifera*, inhibit xanthine oxidase (XOD) and have shown potential in treating gout [107]. Research has demonstrated that broussonetyl polysaccharide offers a protective benefit against liver injury caused by acetaminophen (APAP). It can reduce liver cell apoptosis, enhance antioxidant capacity, improve APAP detoxification ability, and alleviate APAP-induced intestinal flora disorders [110]. In 2021, the antiosteoclast activity of broussonols F, G, and K suggested that these compounds may serve as lead compounds for bone disease treatment, as they significantly inhibited nuclear factor kappa B ligand receptor activator-related osteoclast formation in RAW264.7 cells at doses of 10–30 µM.

Acetylcholinesterase is crucial for cholinergic transmission as it aids in the decomposition of acetylcholine. The ethanol extract derived from *B. papyrifera* includes pentylated flavonoids, which demonstrate inhibitory properties against both human acetylcholinesterase and butyrylcholinesterase, indicating its potential for Alzheimer’s disease therapy [107].

In 2019, Kim et al. conducted in vitro and in vivo experiments and demonstrated that kazinol U, a component found in the root bark of *B. papyrifera*, effectively suppresses the expression of MITF at a dosage of 0–20 µM. This inhibition leads to the activation of the AMPK and MAPK proteins, resulting in the reduction in tyrosinase, Tyrp 1, and Tyrp2 and subsequent melanin formation. Additionally, kazinol U can maintain skin collagen content by neutralizing reactive oxygen species and preventing the activity of collagenase [111].

## 6. Conclusions and Prospects

To advance the research and development of *Broussonetia* flavonoids, this article provides an extensive review of the genes and extraction methods associated with the secondary metabolism of these compounds. It thoroughly organizes both traditional and novel extraction techniques, as well as the pharmacological properties of *Broussonetia* flavonoids and the progress made in applied research within this field.

The pharmacological effects of *B. papyrifera*, including antitumor, antioxidant, anti-inflammatory, antidiabetic, antibacterial, and antiviral activities, are well documented. However, further studies are needed to explore other active compounds and their mechanisms. Investigations reveal that active compounds primarily function by promoting apoptosis, modulating the cell cycle, and inhibiting inflammatory and diabetic pathways. Four key structural genes and eight transcription factors involved in flavonoid biosynthesis have been identified, providing insights into metabolic regulation. Future research should validate the functions of these genes and examine environmental factors influencing flavonoid production. Advances in these areas will enhance the understanding of flavonoid synthesis and support the discovery of novel therapeutic applications.

The extraction and identification of active components from *B. papyrifera* are challenging. Its secondary metabolites are diverse and complex in composition, making the processes of extraction, separation, and identification time-consuming and technically demanding. Currently, the lack of efficient and low-cost separation and purification methods limits the acquisition of single compounds and further research. Additionally, the study of components and their mechanisms of action is insufficient. The interactions between the active components of *B. papyrifera* and multiple targets are complex, and their specific mechanisms of action have not been fully elucidated. Furthermore, the development and conservation of germplasm resources are inadequate. Although *B. papyrifera* has abundant germplasm resources, the development of wild resources is insufficient, and research on variety improvement is limited. Strategies to enhance the medicinal component content require further investigation. *B. papyrifera* extracts (especially flavonoids and isoprene flavonoids) have shown a variety of pharmacological activity in in vitro and in vivo research, including antitumor, anti-inflammatory, antioxidant, and immune regulation. This activity provides a scientific basis for the development of drugs or functional products. As people’s interest in natural products and plant drugs increases, *B. papyrifera* extracts as biologically active substances of natural sources have greater market potential, especially in the field of anticancer, anti-inflammatory, and health care. However, the development of its development needs to overcome obstacles in costs, regulations, and security. By developing, optimizing production technology, and strengthening cooperation in stages, risks can be reduced, and their commercialization process can be accelerated.

## Figures and Tables

**Table 1:** Polyphenolic compounds found in plants of *B. papyrifera*.

No.	Compound Name	Source	Classification	Reference
1	Broussonin A	Leaves	Chalcones	[24]
2	Moracin N	Whole plants	Isoflavonoids	[24]
3	Demethylmoracin I	Whole plants	Isoflavonoids	[24]
4	1-(2,4-Dihydroxyphenyl)-3-(4-hydroxyphenyl)-propane	Whole plants	Diphenylpropane polyphenol	[24]
5	Albanol A	Whole plants	Chalcone- Isoflavonoids	[24]
6	Broussonin B	Roots	Chalcones	[25]
7	Kazinol F	Whole plants	Lignans	[25]
8	Kazinol C	Roots\Twigs	Lignans	[25]
9	Kazinol D	Roots\Twigs	Lignans	[25]
10	Curculigoside C	Fruits	Phenylethanoid Glycosides	[22]
11	3,4-Dihydroxybenzoic acid	Fruits	Phenolic Acids	[22]
12	(7R,8S) -3-Methoxy-4,9,9-trihydroxy-4,7-epoxy-5,8-neolignan	Whole plants	Lignans	[26]
13	(7R,8S,8R)-7,8-Threo-3-methoxy-7-oxo-4,4,7,9,9-pentahydroxy-4,8:7,9-bis-epoxy-8,8-sesquineolignan	Fruits	Lignans	[22]
14	Kazinol W	Roots	Lignans	[26]
15	Kazinol J	Roots\Leaves	Lignans	[26]
16	Kazinol V	Roots	Lignans	[26]
17	Broussonin E	Roots	Lignans	[26]
18	Broussoside D	Leaves	Lignans	[26]
19	3,5,4-Trihydroxy-bibenzyl-3-O-β-D-glucoside	Leaves	Lignans	[26]
20	Broussofluorenone A	Roots	Benzopyrone polyphenols	[26]
21	Moracin I	Whole plants	Lignans	[26]
22	Moracin D	Whole plants	Lignans	[26]
23	Moracin M	Whole plants	Lignans	[26]
24	Altertoxin I	Whole plants	Furanone polyphenols	[24]
25	Altertoxin IV	Whole plants	Furanone polyphenols	[24]
26	Erythro-1-(4-hydroxyphenyl)-2-{4-[(E)-3-hydroxy-1-propenyl]-2-methoxyphenoxy}-1,3-propanediol	Whole plants	Polyphenolic	[27]
27	erythro-1-(4-hydroxyphenyl)-2-[4-(3-hydroxy-1-propyl)-2-methoxyphenoxy]-1,3-propanediol	Whole plants	Polyphenolic	[27]
28	Threo-1-(4-hydroxyphenyl)-2-{4-[(E)-3-hydroxy1-propenyl]-2-methoxyphenoxy}-1,3-propanediol	Whole plants	Polyphenolic	[27]
29	threo-1-(4-hydroxyphenyl)-2-[4-(3-hydroxy-1-propyl)-2-methoxyphenoxy]-1,3-propanediol	Whole plants	Polyphenolic	[27]

**Table 2:** Phenylpropanoids found in plants of *B. papyrifera*.

No.	Compound Name	Source	Classification	Reference
1	Chushizisin A	Fruits	Flavonols	[29]
2	Chushizisin B	Fruits	Flavonols	[29]
3	Chushizisin C	Fruits	Flavonols	[29]
4	Chushizisin D	Fruits	Flavonols	[29]
5	Chushizisin E	Fruits	Flavonols	[29]
6	Chushizisin F	Fruits	Flavonols	[29]
7	Chushizisin G	Fruits	Flavonols	[29]
8	Chushizisin H	Fruits	Flavonols	[29]
9	Chushizisin I	Fruits	Flavonols	[29]
10	Threo-1-(4-hydroxy-3-methoxyphenyl)-2-{4-(E)-3-hydroxy-1-propenyl-2-methoxyphenoxy}-1,3-propanediol	Fruits	Phenolics	[29]
11	Erythro-1-(4-hydroxy-3-methoxyphenyl)-2-{4-(E)-3-hydroxy-1-propenyl-2-methoxy-phenoxy}-1,3-propanediol	Fruits	Phenolics	[29]
12	3-2-(4-Hydroxyphenyl)-3-hydroxymethyl-2,3-dihydro-1-benzofuran-5-ylpropan-1-ol	Fruits	Phenolics	[29]
13	*p*-Coumaraldehyde	Fruits	Phenolics	[22]
14	Cissyringin	Fruits	Phenolics	[22]
15	Cisconiferin	Fruits	Phenolics	[22]
16	Erythro-1-(4-hydroxyphenyl) glycerol	Fruits	Phenolics	[22]
17	Threo-1-(4-hydroxyphenyl) glycerol	Fruits	Phenolics	[22]
18	Dihydroconiferyl alcohol	Fruits	Phenylpropanoids	[22]
19	Dihydrosyringin	Leaves	Phenylpropanoids	[30]
20	Syringaresinol-4-O-β-D-glucoside	Leaves	Lignans	[30]
21	Pinoresinol-4-O-β-D-glucopyranoside	Leaves	Lignans	[30]
22	*p*-Coumaric acid	Leaves	Phenylpropanoids	[30]
23	6,7-Dimethoxycoumarin	Whole plants	Coumarins	[27]
24	Iariciresinol-9-O-β-D-glucopyranoside	Leaves	Lignans	[27]
25	3,4,5-Trihy- droxy-5-methoxy-6H-benzo [c] chromen-6-one	Whole plants	Phenylpropanoids	[27]
26	Alternariol-4-O-methyl ether	Whole plants	Phenylpropanoids	[27]
27	Alternariol-5-O-methyl ether	Whole plants	Phenylpropanoids	[27]
28	(S)-Marmesin	Twigs	Phenylpropanoids	[31]
29	(S)-8-Methoxymarmesin	Twigs	Phenylpropanoids	[31]
30	7,8-Dihydroxy-6-(3-methylbut-2-en-1yl)-2H-chromen-2-one	Roots	Phenylpropanoids	[28]
31	(+)-Pinoresinol-4-O-β-D-glucopyranosyl-4-O-β-D-apiofuranoside	Leaves	Phenylpropanoids	[28]

**Table 3:** Terpenoids found in plants of *B. papyrifera*.

No.	Compound Name	Source	Reference
1	Broussonetone A	Leaves	[37]
2	Broussonetone B	Leaves	[37]
3	Broussonetone C	Leaves	[37]
4	Taraxerol acetate	Leaves	[37]
5	Squalene	Roots/Fruits	[38]
6	Butyrospermol acetate	Whole plants	[38]
7	Augustic acid	Whole plants	[27]
8	Oleanolic acid	Roots	[27]
9	Lupeol acetate	Whole plants	[27]
10	3β-Acetoxy-tirucalla-7-en-24S,25-diol	Roots	[27]
11	(3β)-3-(Acetyloxy)-eupha-7,25-dien-24-one	Roots	[27]
12	(3β,24R)-3-(Acetyloxy)-eupha-7,25-dien-24-ol	Roots	[27]
13	(3β,24S)-Eupha-7,25-diene-3,24-diol	Roots	[27]
14	(3β,24R)-Eupha-7,25-diene3,24-diol	Roots	[36]
15	α-Amyrin acetate	Roots	[36]
16	β-Amyrin	Roots	[36]
17	Lupeol	Roots	[36]

**Table 4:** Alkaloids found in plants of *B. papyrifera*.

No.	Compound Name	Source	Reference
1	Broussonpapyrine	Fruits	[43]
2	Liriodenine	Fruits	[43]
3	Oxyavicine	Fruits	[43]
4	Nitidine	Fruits	[43]
5	Dihydrosanguinarine	Fruits	[43]
6	N-Norchelerythrine	Fruits	[43]
7	2-Deoxyuridine	Whole plants	[28]
8	2-Deoxyadenosine	Whole plants	[28]
9	Thymidine	Whole plants	[28]
10	Isoterihanine	Whole plants	[43]
11	Chelerythrine	Whole plants	[43]
12	Erythrinasinate	Roots	[43]

**Table 5:** Other compounds found in plants of *B. papyrifera*.

No.	Compound Name	Source	Classification	Reference
1	Fucosterol	Whole plants	Sterol	[43]
2	Ergosterol peroxide	Whole plants	Steroid peroxide	[43]
3	β-Sitosterol	Whole plants	Phytosterol	[30]
4	β-Daucosterol	Whole plants	Sterol	[30]
5	Ergosta-4,6,8,22-tetraen-3-one	Whole plants	Steroid	[30]
6	D-Galacitol	Whole plants	Sugar alcohol	[30]
7	Daucosterol palmitate	Whole plants	Sterol ester	[27]
8	Palmitic acid	Whole plants	Saturated fatty acid	[27]
9	Phytol	Whole plants	Carotenoid derivative	[27]
10	Physcion	Whole plants	Anthraquinone	[27]
11	Palmitic acid ethyl ester	Whole plants	Fatty acid ester	[27]
12	Linoleic acid	Whole plants	Polyunsaturated fatty acid	[27]
13	8,11-Octadecadienoic acid	Whole plants	Polyunsaturated fatty acid	[27]
14	9-Octadecenoic acid	Whole plants	Monounsaturated fatty acid	[27]
15	α-Monopalmitin	Whole plants	Glyceride	[27]
16	Monoheptadecanoin	Whole plants	Glyceride	[27]
17	Heptadecanoic acid	Whole plants	Saturated fatty acid	[27]
18	Altersolanol A	Whole plants	Phenolic quinone	[27]
19	Altersolanol C	Whole plants	Phenolic quinone	[27]
20	δ-Tocopherol	Whole plants	Tocopherol	[27]
21	(4R,5S,10S)-8,9,10-Trihydroxy-4-[3-methoxy-4-hydroxyphenyl]-1,6-dioxaspiro [4,5]decan-2-one	Whole plants	heterocyclic compound	[27]
22	4-Hydroxyacetophenone	Whole plants	Phenolic	[27]
23	(7R,8S)-3-Methoxy-7-oxo-4,9,9-trihydroxy-4,7-epoxy-5,8-neolignan	Whole plants	Neolignans	[27]
24	(7R,8S)-3-Methoxy-4,9,9-trihydroxy-4,7-epoxy-5,8-neolignan	Whole plants	Neolignans	[27]
25	Benzylbenzoate-2,6-di-O-β-D-glucopyranoside	Whole plants	Benzylbenzoate	[27]
26	Broussoside A	Leaves	Phenylethanoid	[30]
27	Broussoside C	Leaves	Phenylethanoid	[30]
28	Broussoside E	Leaves	Phenylethanoid	[30]
29	Poliothyrsoside	Leaves	Phenylethanoid	[30]
30	4-Hydroxybenzaldehyde	Fruits	Aromatic aldehydes	[22]
31	Curculigoside I	Fruits	Phenylethanoid	[22]
32	2-(4-Hydroxyphenyl) propane-1,3-diol-1-O-β-D-glucopyranoside	Fruits	Phenylpropanoid	[22]
33	(2R,3R,5R,6S,9R)-3-Hydroxy-5,6-epoxyb-ionol-2-O-β-D-glucopyranoside	Leaves	Carotenoid derivative	[22]
34	Lignoceric acid	Roots	Saturated fatty acid	[43]
35	Octacosan-1-ol	Roots	long-chain alcohol	[43]
36	4-Hydroxycis-cinnamic acid octacosyl ester	Roots	Cinnamic acid ester	[43]

**Table 6:** Flavonoids found in plants of *B. papyrifera*.

No.	Compound Name	Source	Classification	Reference
1	Papyriflavonol A	Roots\Twigs	Flavonoids	[28]
2	5,7,3′,4′-Tetrahydroxy-6,5′-di-(γ,γ-dimethylallyl)-flavonol	Leaves	Flavonols	[28]
3	8-(1,1-Dimethylallyl)-5′-(3 methylbut-2-enyl)-3′,4′,5,7-tetrahydroxyflavonol	Barks	Flavonols	[27]
4	3′-(3-Methylbut-2-enyl)-3′,4′,7-trihydroxyflavane	Barks	Flavans	[27]
5	5,7,3′,4′-Tetrahydroxy-3-methoxy-8-ger anylflavone	Barks	Flavonoids	[31]
6	5,7,3′,4′-Tetrahydroxy-3-me thoxy-8,5′-diprenylflavone	Barks	Flavonoids	[31]
7	3,5,7,4′-Tetrahydroxy- 3′-(2-hydroxy-3-methylbut-3-enyl	Barks	Flavonoids	[31]
8	Quercetin	Twigs	Flavonols	[43]
9	Dihydroquercetin	Whole plants	Flavonols	[43]
10	Isolicoflavonol	Whole plants	Flavonols	[43]
11	Broussoflavonol G	Whole plants	Flavonols	[43]
12	Broussoflavonol F	Roots\Twigs	Flavonols	[43]
13	Broussoflavonol E	Roots\Twigs	Flavonols	[43]
14	4′7-Dihydroxyflavone	Leaves	Flavonoids	[28]
15	Broussonol A	Leaves	Flavonols	[43]
16	Broussonol B	Leaves	Flavonols	[43]
17	Broussonol C	Roots\Leaves	Flavonols	[43]
18	Broussonol D	Roots\Leaves	Flavonols	[43]
19	Broussonol E	Twigs	Flavonols	[43]
20	Apigenin	Leaves	Flavonols	[43]
21	Cosmosiin	Leaves	Flavonoids	[43]
22	Luteolin	Leaves\Twigs	Flavonoids	[43]
23	Luteolin-7-O-β-D-glucopyranoside	Leaves	Flavonoids	[43]
24	7-Methoxyapigenin	Leaves	Flavonoids	[43]
25	5,7,3′,4′-Tetrahydroxy-3-methoxy-6-geranylflavone	Barks	Flavonoids	[43]
26	5,7,2′,4′-Tetrahydroxy-3-geranylflavone	Barks	Flavonoids	[43]
27	(2S)-Abyssinone II	Whole plants	Flavonoids	[43]
28	(2S)-5,7,2′4′-Tetrahydroxyflavanone	Barks	Flavans	[43]
29	Broussochalcones A	Whole plants	Chalcones	[43]
30	Broussochalcones B	Whole plants	Chalcones	[43]
31	3′,4′,7-Trihydroxyfiavone	Barks	Flavonoids	[43]
32	Formononetin	Leaves\Twigs	Isoflavones	[43]
33	Epiafzelechin	Leaves\Twigs	Flavanols	[43]
34	Arbutin	Whole plants	Flavonoids	[43]
35	Broussoside B	Whole plants	Flavonoids	[43]
36	Flacourtin	Leaves	Flavonols	[30]
